# Isotropic Hyperfine Interactions Drive Cross-Effect Dynamic Nuclear Polarization

**DOI:** 10.1021/acs.jpclett.5c02845

**Published:** 2025-11-07

**Authors:** Nitzan Livni, Subhradip Paul, Ilia B. Moroz, Alexey V. Bogdanov, Daniel Jardón-Álvarez, Frederic Mentink-Vigier, Michal Leskes

**Affiliations:** † Chemical and Biological Physics, 34976 Weizmann institute of science, Rehovot, 761000, Israel; ‡ Nottingham DNP MAS NMR Facility, University of Nottingham, Nottingham NG72RD England, U.K.; § Univ. Grenoble Alpes, CEA, IRIG-MEM, 38000 Grenoble, France; ∥ National High Magnetic Field Laboratory, Florida State University, 1800 E Paul Dr, Tallahassee, Florida 32310, United States

## Abstract

Dynamic nuclear polarization (DNP) is a powerful route for overcoming the inherent sensitivity limitation of solid-state nuclear magnetic resonance (ssNMR) spectroscopy by transferring high electron spin polarization to surrounding nuclear spins. Cross-effect (CE) DNP is the most efficient mechanism in solids. CE requires several conditions to be met, primarily the presence of two coupled electron spins with resonance frequencies separated by the nuclear Larmor frequency. This condition is typically achieved through the presence of large anisotropic spin interactions, which shift the transition frequencies of the two coupled electron spins with respect to each other. Here we present an alternative approach, where the CE condition is met via isotropic interactions. This is advantageous as it makes CE independent of the sample orientation, thus making the enhancements independent of the MAS frequency and enabling the use of fast relaxing polarizing agents. We demonstrate the feasibility of the approach in experiments and simulations for Mn­(II) dopants as polarizing agents, making use of the isotropic hyperfine interactions with its ^55^Mn nuclear spin to achieve the required frequency difference.

## Graphical abstract

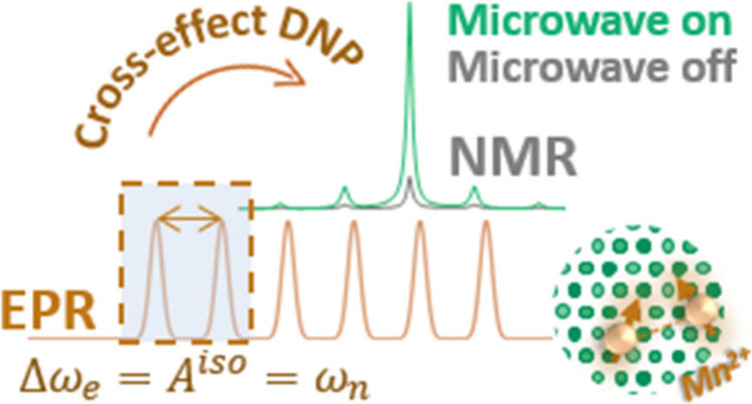

Solid-state nuclear magnetic resonance (ssNMR) spectroscopy is a powerful method for obtaining atomic scale information on solids.^1^ Yet, the limited nuclear spin polarization and low abundance of NMR-active isotopes lead to inherent sensitivity constraints, which restrict the applicability of this highly informative technique. Dynamic nuclear polarization (DNP) has transformed the landscape of ssNMR.^2^ DNP utilizes the substantial polarization of electron spins and channels it to the observed coupled nuclear spins by applying microwave irradiation at specific frequencies to saturate electronic spin transitions.

Among the various DNP mechanisms, cross-effect DNP (CE-DNP) stands out as exceptionally efficient in solid materials, delivering up to 10^4^-fold enhancement in NMR sensitivity.^3–6^ The simplest model for describing this mechanism includes three spins: two coupled electron spins and one nuclear spin, coupled to one of the electrons. This simplified system can be described by a Hamiltonian which contains the Zeeman interactions ( $H_{z}^{i}$ ) of each spin and the couplings between them ( $H_{D}^{ij}$ , full Hamiltonian terms are shown in the Supporting Information):

1 $$H_{0}=H_{z}^{e_{1}}+H_{z}^{e_{2}}+H_{z}^{n}+H_{D}^{en}+H_{D}^{ee}$$

For the transfer of polarization via the CE mechanism, the difference in energy levels of the two coupled electron spins, Δ*ω*_*ee*_=|ω_*e*_1__ – ω_*e*_2__|, must be equal to the nuclear Larmor frequency: Δ*ω*_*ee*_ = |*ω*_*n*_|. This condition leads to a degeneracy between the flip-flop-flip Zeeman eigenstates (|αβα⟩ and |βαβ⟩ or |αββ⟩ and |βαα⟩). The degeneracy results in efficient mixing of the energy states, thereby driving spin population transfer through electron–electron and electron–nuclear dipolar coupling terms. When there is an imbalance in polarization between the two electron spins Δ*p*_*ee*_, it can result in polarization buildup across the nuclear spin transitions.^7^ We can substantially increase Δ*p*_*ee*_ by selectively saturating one of the single-quantum (SQ) electron spin transitions using microwave irradiation. The combination of high Δ*p*_*ee*_ along with the fulfillment of the CE degeneracy condition and the presence of significant spin couplings results in pronounced hyperpolarization of the nuclear spin.

Commonly, the polarizing agents used for CE-DNP are carefully designed and optimized organic biradicals.^8–12^ In these molecules, the g-anisotropy leads to the difference in the electron spin resonance frequency, which is necessary for satisfying the CE condition. This is achieved either by coupling of two different radicals or coupling the same type of radical bound with different orientation with respect to the magnetic field (Figure 1a). A very successful class of polarizing agents is based on nitroxide biradicals, which were optimized for delivering CE-DNP. The tilt in the relative g-tensors between the two radicals increases the probability for matching the CE condition in this strongly coupled two-electron spin system.^13^ Other types of anisotropic interactions can also yield DNP enhancement through the CE mechanism. Kaushik et al. showed that paramagnetic high spin metal ion complexes, where the zero field splitting (ZFS) is the source of anisotropy and frequency difference between metal centers, can lead to enhancement via CE-DNP.^14^

**Figure 1:**
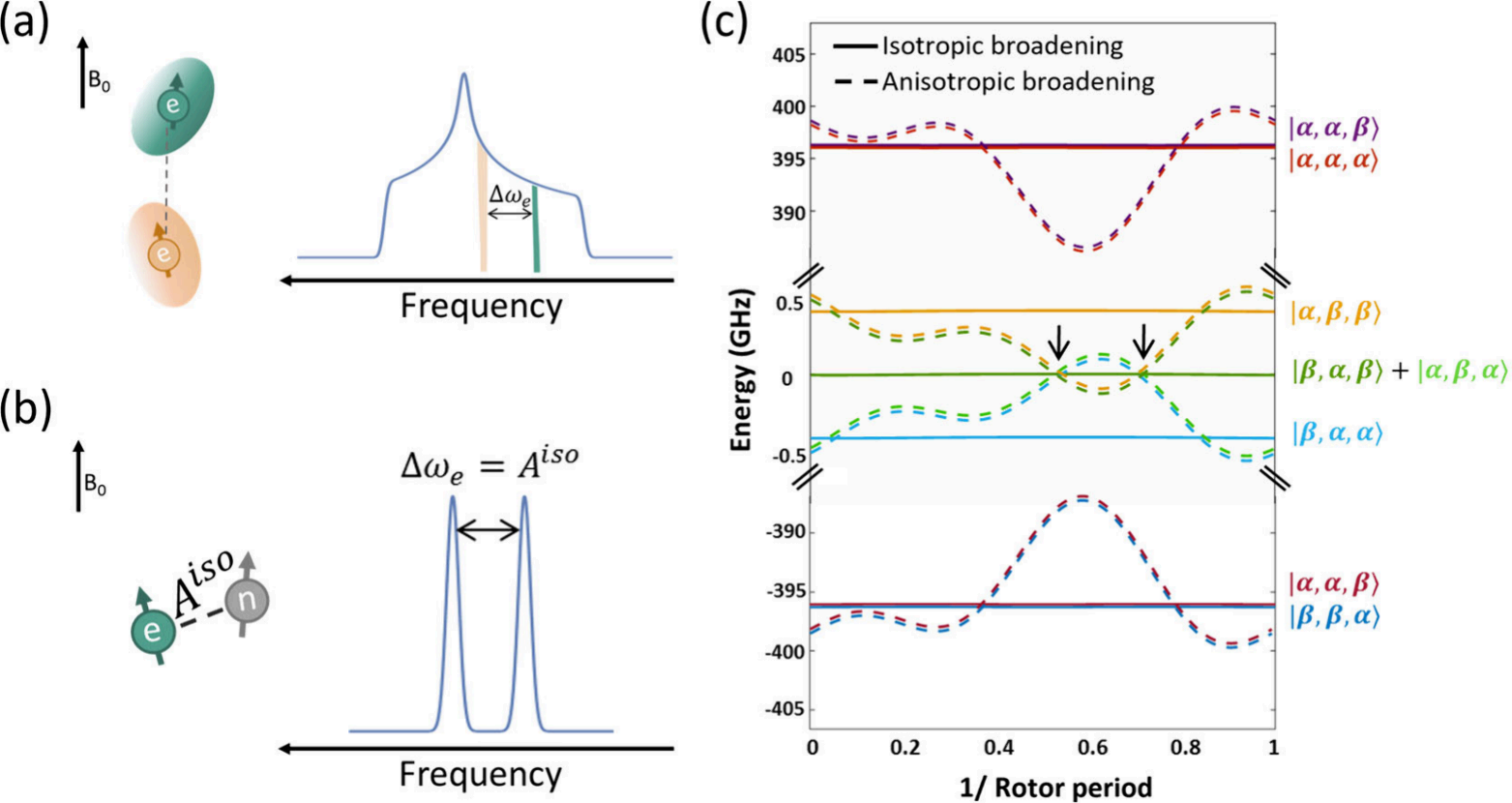
(a) Illustration of an EPR spectrum broadened by anisotropic spin interactions. The anisotropic interaction creates a distribution of frequencies between individual electron spin resonances arising from different orientations with respect to the magnetic field. (b) Isotropic hyperfine coupling shifts the electron spin energy levels, resulting in a splitting in the EPR profile given by the strength of the hyperfine coupling. (c) Under MAS, the anisotropic interactions induce shifts in the energy levels. In this case, the CE conditions will be satisfied at specific times within a rotor period (marked with arrows). Conversely, in an isotropic-CE system, the splitting of the energy levels is independent of the orientation, ensuring the continuous matching of the CE condition independent of MAS.

In practice, as most ssNMR experiments are done in combination with magic angle spinning (MAS) to increase the spectral resolution, the process of polarization transfer via CE is more complex. Under MAS, the sample orientation relative to the external magnetic field leads to a periodic oscillation of the energy levels derived by the anisotropic interactions (Figure 1c, dashed lines). As a result, under spinning, a broad range of electron spins with different orientations can satisfy the CE conditions at different times. Furthermore, under MAS the CE process breaks into a series of consecutive events — saturation of one of the electron spins SQ transitions, mixing of energy states by dipolar interactions, and the transfer of polarization to the nucleus. The polarization is accumulated via this cycle of “rotor events” with efficiency that depends on the rate of change in the magnitude of the different anisotropic interactions at each event.^15–17^ Often, this leads to unfavorable MAS dependence of the CE mechanism.^16,18,19^ Furthermore, the stepwise nature of the CE mechanism under MAS results in efficient DNP only for relatively slow (relative to a rotor period) relaxing electron spins, since the system must maintain its state between rotor events to enable effective accumulation of nuclear polarization.

An alternative, MAS-independent route for CE—compatible with both fast MAS NMR measurements and rapidly relaxing polarizing agents—would require a system in which the electron resonances are separated by an isotropic interaction (Figure 1b).^20^ In this case, if the CE conditions are met, the degeneracy of energy levels occurs continuously (Figure 1c, continuous lines). In this work, we demonstrate this concept in metal-ion DNP (MIDNP) by utilizing the isotropic hyperfine interactions of Mn­(II) (S = 5/2) with its own nuclear spin ^55^Mn (I = 5/2). MIDNP is commonly applied in inorganic solids, where paramagnetic metal ions are introduced as dopants and used as endogenous polarization agents.^21^ In inorganic solids, MIDNP is advantageous, as it enables efficient polarization transfer in the bulk, even in the absence of efficient spin diffusion, which is often the case in the absence of protons. Instead, polarization is transferred directly from the metal ions, providing sensitivity for detecting low natural abundance nuclear spins in the bulk and surface of the solid.^22–26^ To date, the most common DNP mechanism used in MIDNP is the solid effect (SE) that facilitates the transfer of electron spin polarization through the saturation of nominally forbidden zero-quantum (ZQ) and double-quantum (DQ) spin transitions. As such, it is less efficient than the CE mechanism. While CE matching conditions can be achieved in metal-doped systems via anisotropic interactions—such as strong electron–electron dipolar coupling or ZFS—it has not yet proven to be efficient in MIDNP.^14,27^ One limiting factor is the short longitudinal electron spin relaxation time (T_1__e_) of high-spin metal ions (of the order of 10^–7^–10^–5^ seconds), which hinders the accumulation of polarization over multiple rotor periods (typically 10^–5^–10^–4^ seconds). In future work, we will explore the scope and constraints of CE via anisotropic interactions in common MIDNP systems. Nonetheless, enabling CE through isotropic interactions, which would render the process almost MAS-independent (as dipolar and ZFS interactions may still lead to some MAS dependence) and potentially alleviate the reliance on long electron relaxation times, represents a promising strategy to enhance both CE efficiency and overall NMR sensitivity.

Here, we demonstrate this approach in two systems where the CE condition is approached by matching the Mn­(II) hyperfine interaction, A^iso^, introduced as a dopant, and the Larmor frequency (*ω*_*n*_) of one of the nuclei in crystalline powders. We first show, through the DNP sweep profile, the contribution of the CE mechanism and its differentiation from the contribution of the SE. Next, we investigate the relation between different experimental parameters and CE efficiency, including MAS. Finally, we use numerical simulations and electron–nuclear double resonance (ENDOR) measurements to understand the factors limiting the CE efficiency in these systems.

A typical Mn­(II) EPR spectrum (Figure 2b and Supporting Information) displays the characteristic splitting of the central electron spin transition (|−1/2⟩ ↔ |+1/2⟩) into six resonances arising from the isotropic hyperfine interaction. When Mn­(II) ions are incorporated into sites with high local symmetry in crystalline solids (such as perfectly tetrahedral or octahedral sites), it exhibits negligible g-anisotropy. In addition, at high magnetic fields (*ω*_*e*_ ≫ *ZFS*), as in high field DNP experiments, the central electron transition is relatively unaffected by the ZFS.^21^ Consequently, the six lines originating from the hyperfine couplings are narrow with line widths on the order of 10^–3^ Tesla. The main sources of spectral broadening of the hyperfine sextet are transverse relaxation, T_2e_, and electron–electron dipolar couplings. The frequency difference between the isotropic electron resonances, ranging between 170 and 280 MHz, depending on the Mn coordination environment, provides several possibilities to meet the CE condition for different nuclei at different magnetic fields.^28^ This makes Mn­(II) a promising choice as a polarizing agent for realizing isotropic-interaction-driven CE. We examine the feasibility for an isotropic-CE in two systems doped with Mn­(II): Li_4_Ti_5_O_12_ (LTO) and Na_2_ZnP_2_O_7_ (NZPO, see Supporting Information for details about synthesis and characterization). Field sweep echo detected (FSED) EPR spectra were acquired for both compounds on a W-band (94 GHz; Figure S3). The spectra were fitted, allowing to determine A^iso^ = 229 and 245 MHz for LTO and NZPO, respectively. These parameters closely match the Larmor frequencies of ^7^Li and ^31^P at 14.1 T, *ω*_*n*_(^7^Li) = 233 MHz, *ω*_*n*_ (^31^P) = 243 MHz, approaching an ideal fit to the CE condition.

**Figure 2:**
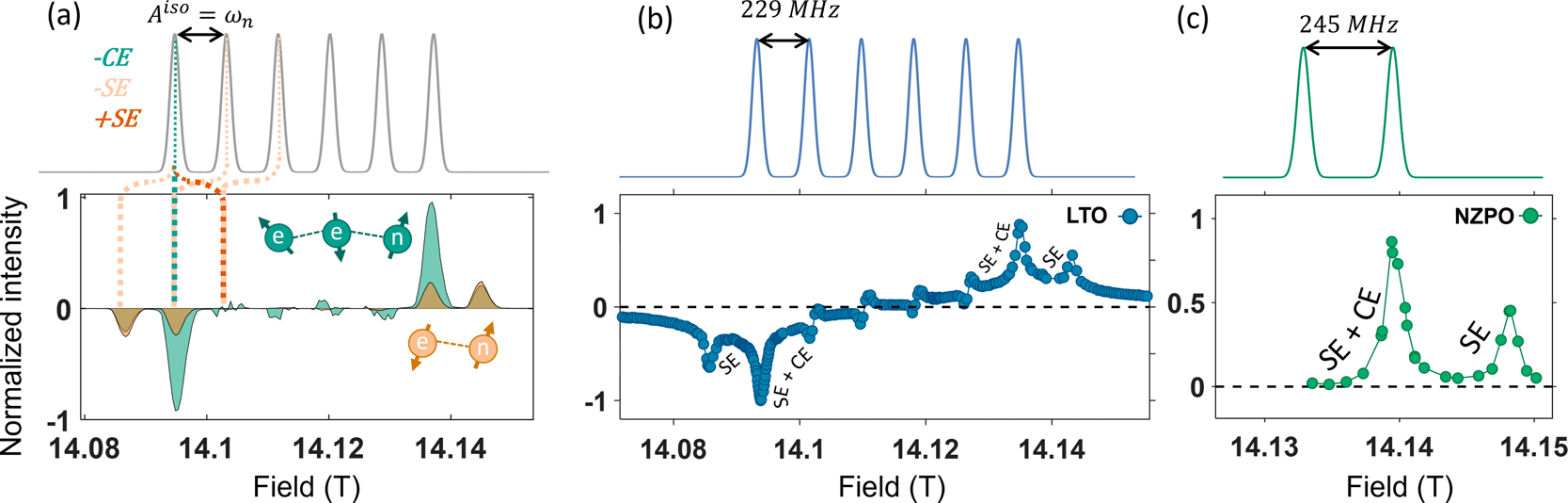
(a) Simulated DNP field sweep profile for a simplified two (orange) or three (green) spin system, including one (orange) or two (green) electrons and one coupled nuclear spin. The simulated Mn­(II) EPR profile is plotted above. The dashed lines mark how each EPR transitions translates to the sweep profile for different DNP mechanisms. Experimental DNP field sweep profiles measured for: (b) ^7^Li in 40 mM Mn­(II) doped LTO and (c) ^31^P in 40 mM Mn­(II) doped NZPO. The corresponding simulated high-field EPR spectra are plotted above.

EasySpin^29^ simulation of a typical Mn­(II) central transition EPR profile (top) and the corresponding DNP field sweep profile (bottom) is shown in Figure 2a for a simplified system containing a single Mn­(II) dopant or two coupled dopants and a nucleus with A^iso^ = *ω*_*n*_. The sweep profile shows the NMR signal intensity of the hyperpolarized nuclear spin as a function of the magnetic field acquired with fixed frequency microwave irradiation. The field sweep was simulated by adapting a code for calculating DNP from nitroxide radicals and adjusting it to our system.^30^ We simplified the calculation by considering the Mn­(II) electrons as spins 12 , under the assumption that the central transition predominantly contributes to the DNP process and neglecting the effect of ZFS. In addition, if we assume that the ^55^Mn nuclei do not contribute to the mechanism, then we can avoid the lengthy calculation of a time-dependent 5-spins system (2 coupled electrons with strong isotropic couplings to a nuclear spin and a detectable nucleus coupled to at least one of the electrons via dipolar interactions) necessary for the isotropic-hyperfine driven CE mechanism. The total Hamiltonian can be projected over the nuclear states of ^55^Mn, which form independent subspaces. We used this approximation and simulated the standard three-spin system for CE mechanism replacing the ^55^Mn spin as an additional effective magnetic field affecting only the electron spins:

H0=Hze1+Hze2+Hzn+Hdden+Hddee+mI,1·Aiso·S^z1+mI,2·Aiso·S^z2 2

with $m_{I,i}=\pm \left[\frac{1}{2},\frac{3}{2},\frac{5}{2}\right]$ assigned randomly for each electron in the system. The whole calculation is repeated and averaged over a set of crystal orientations to resemble the result of a powder sample. The isotropic hyperfine coupling was set to the experimentally measured value of A^iso^ = 229 MHz for Mn­(II) in LTO and the nuclear Larmor frequency was set to ω_n_ = 233 MHz (*ω*_*n*_(^7^Li) at 14.1 T). The details of the code have been previously described.^20^ The projection procedure together with the entire simulation parameters can be found in the Supporting Information. For a system containing a single electron, the DNP mechanism is the SE, where negative and positive NMR enhancements are obtained when the ZQ/DQ transitions are saturated at ±*ω*_*n*_ from the electron spin SQ transition. The simulated DNP field sweep for a single dopant (Figure 2a, bottom spectrum) reveals that the NMR signal enhancement is observed only for the two outermost transitions. This unique profile is due to the signal cancellations from overlapping SE-DNP at ZQ/DQ transitions from adjacent electron spin transitions for the specific choice of A^iso^ = *ω*_*n*_.

Introducing an additional electron spin into the system enables the CE DNP mechanism. Since CE requires saturation of the electron SQ transition, NMR enhancement is obtained when matching the frequencies of these transitions. The simulated sweep for our two-electron configuration (Figure 2a, bottom) reveals a pattern similar to the single-electron case, with the notable exception of a higher enhancement at the field positions corresponding to the outermost SQ electron transitions. When A^iso^ = *ω*_*n*_, these SQ transitions coincide with the ZQ and DQ transitions of the neighboring electron spin levels. The increased enhancement observed in the two-electron system compared with the single-electron case provides clear evidence for the contribution of the CE mechanism. Simulations of the buildup of polarization as a function of polarization time clearly indicate the presence of two DNP processes at the position of the outermost electron transition within the hyperfine manifold, and a single process at the DQ transition shifted by the nuclear Larmor frequency (Figure S5).

Figure 2b-c presents the experimental DNP sweep profiles obtained at 100 K and 14.1 T for 40 mM Mn­(II)-doped LTO and NZPO at a MAS rate of 8 kHz with microwave power of 14.5 and 16 W for LTO and NZPO, respectively. The profile measured for the LTO sample spans the full range of the Mn­(II) hyperfine transitions (arising from the central electron spin transition), whereas for NZPO the profile captures only the two high-field DNP positions. Both experimental profiles exhibit patterns consistent with the simulated profiles for the two-electron configuration, supporting the realization of the isotropic CE in these systems. Although the contribution of the satellite electron transitions to the DNP mechanism was considered negligible, it can contribute to a broad baseline in the DNP field sweep profile, as observed experimentally for Mn­(II)-doped LTO (Figure 2b).

In order to estimate the contribution of CE to the signal enhancement, we calculate the difference between the enhancement obtained at a field position contributed from both SE and CE mechanisms and the outer peak which corresponds to pure SE mechanism. Here we used the field positions, yielding positive enhancements. First, we define *ε*_SE_ and *S*_SE_ which refer to the enhancement and signal intensity of the highest field peak in the DNP field sweep profile, attributed solely to SE; *S*_μWoff_ is the NMR signal intensity acquired without microwave irradiation. The enhancement factor for the SE mechanism in our systems is calculated as

3 $$\varepsilon_{\mathrm{SE}}=S_{\mathrm{SE}}/S_{\mu Woff}$$

From there we can estimate the CE enhancement

4 $$\varepsilon_{\mathrm{CE}}=\frac{S_{\mathrm{mixed}}}{S_{\mu Woff}}-\varepsilon_{\mathrm{SE}}+1$$

where *S*_mixed_ is the signal intensity of the adjacent positive peak, associated with both DNP mechanisms. We note that this calculation might be a slight underestimation of the CE enhancement since our simulations with different polarization times indicate the two DNP mechanisms can affect each other. Thus, the overall enhancement at a field position where both mechanisms occur is not exactly equal to the contribution of each mechanism independently (Figures S5, S9).

We now turn to examine how different experimental parameters influence the DNP efficiency in our systems, namely, dopant concentration, microwave power, and MAS rate. Increasing the dopant concentration increases the probability of strong couplings between dopants, which should promote the occurrence of the CE. However, higher concentrations can also lead to shortening of electron spin relaxation which could reduce the saturation efficiency and consequently the enhancement.^31^ Finally, higher concentrations can result in loss of NMR signal due to quenching. Figure 3a shows the NMR enhancement factor (*ε*) for the SE and CE mechanisms in the two materials as a function of the dopant concentration (the spectra are shown in Figure S4). For both LTO and NZPO, *ε*_SE_ decreases as the dopant concentration increases, consistent with enhanced relaxation processes. In the LTO system, *ε*_CE_ increases with higher dopant levels, suggesting a more efficient CE mechanism, while for NZPO, no significant changes are observed within the studied range of concentrations.

**Figure 3:**
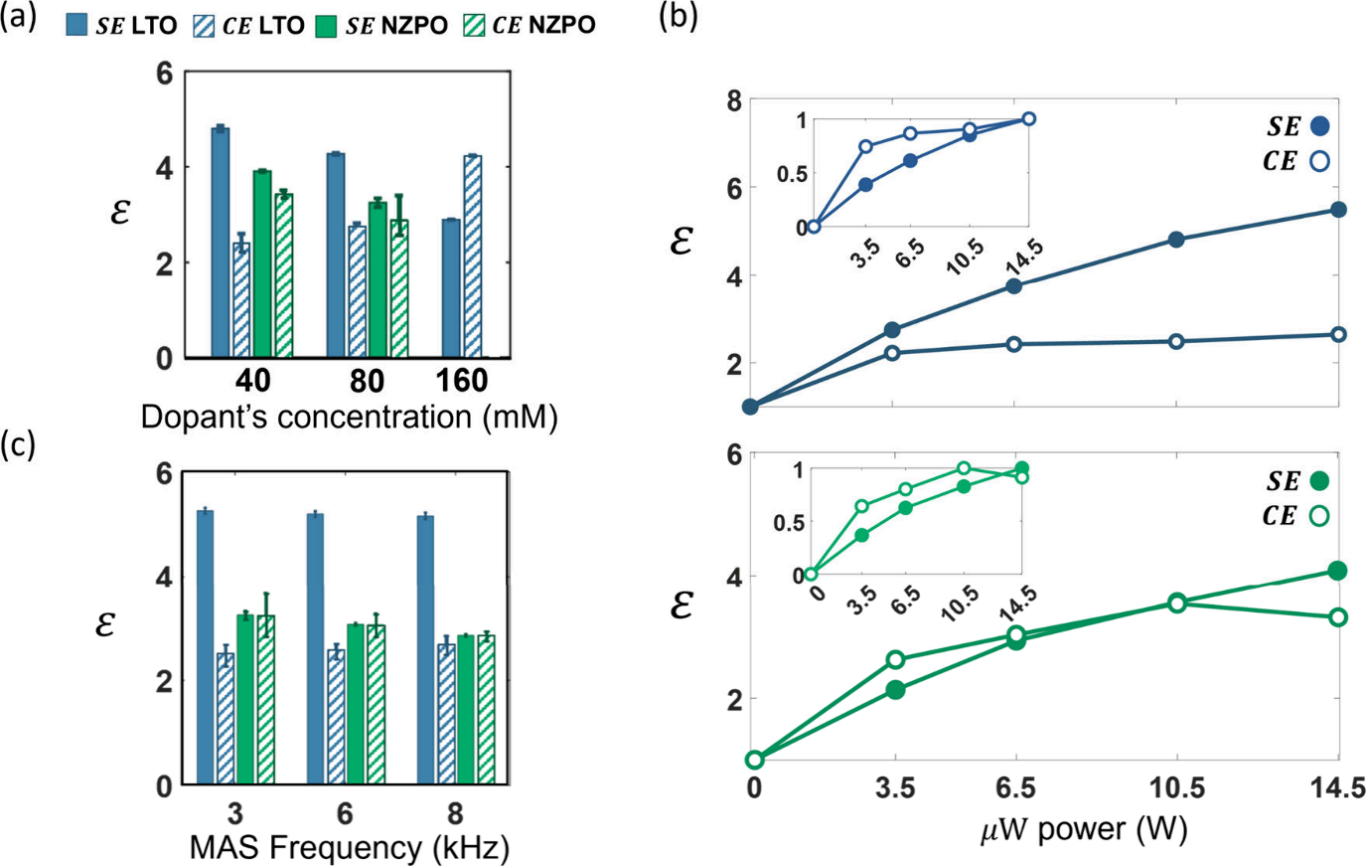
(a) Measured SE (solid) and CE (stripes) DNP signal enhancement for ^7^Li at LTO (blue) and ^31^P at NZPO (green) with different dopant concentrations. (b) Microwave power dependence of the enhancement measured for 40 mM Mn­(II) doped LTO (top, blue) and NZPO (bottom, green), for SE (full) and CE (empty) DNP mechanisms measured at 8 kHz MAS. The normalized data is shown in the inset. (c) MAS dependence of the measured DNP enhancement for SE (solid) and CE (stripes) for ^7^Li at LTO (blue) and ^31^P at NZPO (green). Measurements were carried out on 80 mM Mn­(II) doped samples with 14.2 W microwave power.

We further investigated the dependence of the DNP mechanism efficiency on microwave power. Figure 3b shows the microwave power dependence of the NMR enhancement factors for LTO (top) and NZPO (bottom), with irradiation power ranging from zero to 14.5 W. As previously noted, the SE mechanism involves saturation of nominally forbidden spin transitions; consequently, SE is expected to have stronger dependence on the microwave power compared to CE. Our measurements show that indeed, in both systems, the CE enhancement reaches a plateau already at low irradiation powers, while the SE enhancement continues to increase at the available power range. Nevertheless, in both systems with 40 mM Mn­(II), the SE mechanism provides equal or higher NMR enhancements compared to CE at all power levels.

Finally, we studied the MAS dependence of the two DNP mechanisms by performing DNP-NMR measurements at the positions of the two positive peaks in the sweep profile, varying the MAS frequency in the range of 3–8 kHz. In systems dominated by isotropic interactions with well-matched CE conditions, both SE and CE mechanisms are expected to be MAS-independent. Figure 3c shows the MAS dependence of each mechanism for the two studied systems. In both systems, the two mechanisms display no dependence on the spinning frequency.

Our experimental results provide clear evidence for the presence of the CE mechanism in both metal-ion-doped systems, with the frequency difference between both electrons originating from isotropic interactions. However, surprisingly, the CE mechanism did not yield significantly higher NMR enhancements. The observed enhancements remained in the single-digit range, comparable to those achieved via the pure SE mechanism across all of the tested experimental conditions. Furthermore, measurements of the NMR signal buildup time under microwave irradiation (*T*_bu_) at the field positions corresponding to the mixed and pure SE mechanisms show similar buildup rates (Figure S6). In contrast, numerical simulations of the polarization buildup curve at the two field positions for a single nuclear spin reveal very different time scales for CE and SE mechanisms (Figure S5). The discrepancy between experiments and simulations is likely the presence of spin diffusion in these samples which leads to uniform time scale of polarization transfer. This is consistent with previous studies showing spin diffusion is efficient in natural abundance ^7^Li in LTO^32^ and phosphorus-rich phases.^33^ Consequently, the experimental buildup curves do not contain information regarding the time scale of polarization transfer via different DNP mechanisms that can be seen in the simulated buildup.

To gain a better understanding of the experimental results, we performed numerical simulations to examine different scenarios that can affect the efficiency of the isotropic CE mechanism. We begin with mapping the effect of electron–electron dipolar interactions (ω_*D*_^*ee*^) and electron–nuclear dipolar interactions (ω_*D*_^*en*^). Figure 4a shows examples of simulated partial field sweep profiles of the three spins system (*e*–*e*–*n*), calculated with fixed relaxation parameters and varying electron–electron (*r*_*ee*_) and electron–nuclear (*r*_*en*_) distances. The strength of the dipolar interactions is related to the distance between the spins via:

5 $$\omega_{D}^{ij}=-\frac{\mu_{0}}{4\pi }\cdot \frac{\gamma_{i}\gamma_{j}\hbar }{r^{3}}$$

with γ being the gyromagnetic ratio of the electron or the nucleus (here lithium). The simulations illustrate how the sweep profile (and the EPR line) changes due to the strong anisotropic ω_*D*_^*ee*^ interactions at short *r*_*ee*_. To account for the changing line shape, the contribution of each mechanism was evaluated by integration over the entire lobe: for SE we integrated the profile around 14.143 T, while the contribution of CE was evaluated from the difference in the integral around 14.135 and 14.143 T. The simulation was performed for different configurations varying *r*_*ee*_ and *r*_*en*_ between 9–55 *Å* and 3–150 *Å*, respectively. The contributions to the signal intensity from the two DNP mechanisms are summarized in the heat maps in Figure 4b.

**Figure 4:**
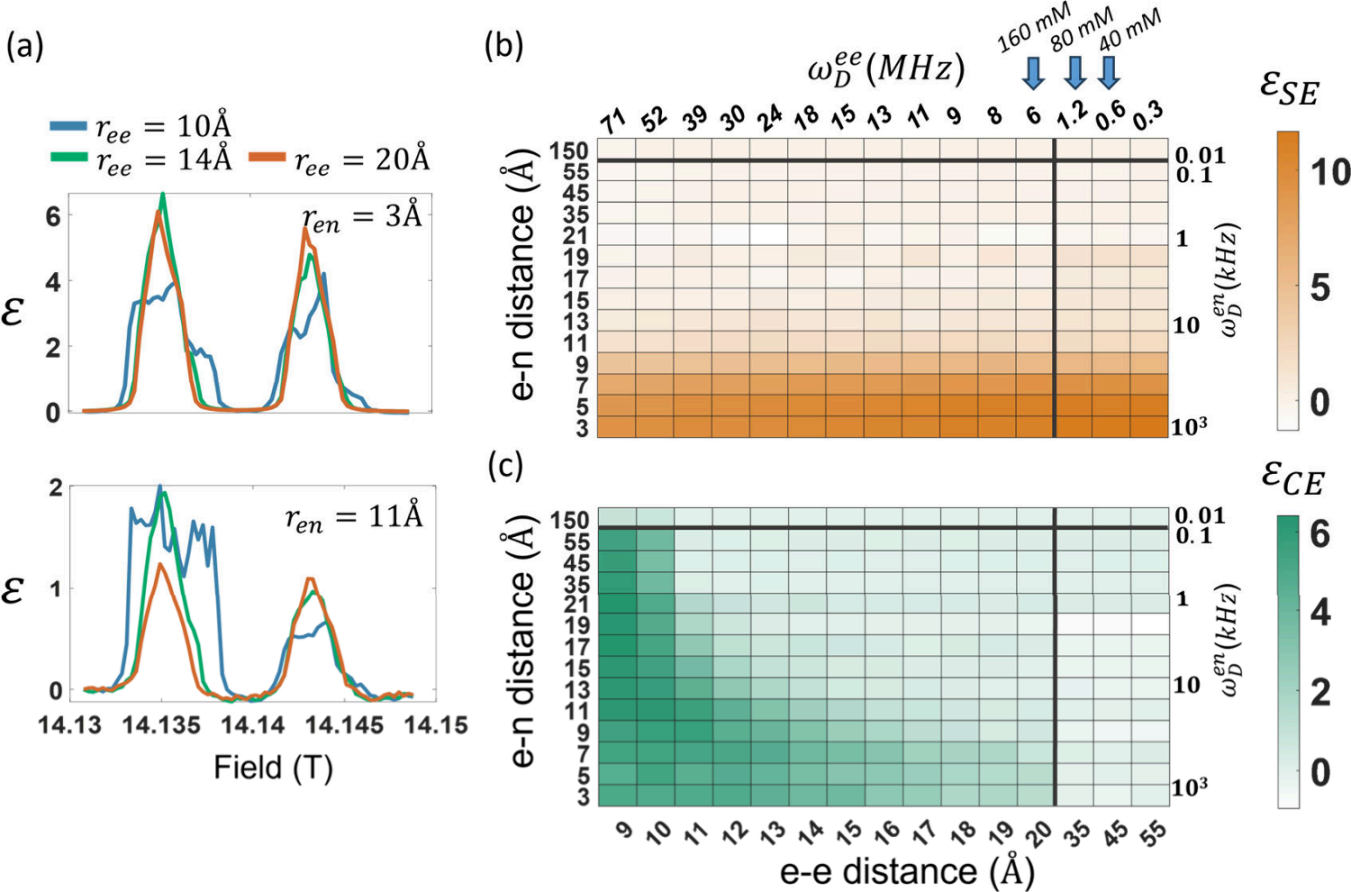
(a) Simulated partial DNP field sweep profile of an e-e-n system with varying electron–electron (*r*_*ee*_) and electron–nuclear (*r*_*en*_) distances and fixed relaxation parameters (*T*_1*e*_ = 1 *μs*, *T*_2*e*_ = 50 *ns*, *T*_1*n*_ = 100 *s*, and *T*_2*n*_ = 1 *ms*). Simulated enhancement factors were obtained for (b) SE and (c) CE mechanisms as a function of the e-e (ω_*D*_^*ee*^) and e-nuclear (ω_*D*_^*en*^) dipolar interactions. SE intensity was calculated from the simulated DNP field sweep profiles as the integral of the peak at 14.143 T. CE intensity was calculated as the difference between the integrals of the peak at 14.135 T and the peak at 14.143 T. Arrows indicate the average *r*_*ee*_ for the dopant concentrations used in experiments, as calculated from the Wigner–Seitz model.

The upper map (orange) shows the intensity attributed to SE as a function of the dipolar couplings. As expected, this mechanism only requires a single electron spin; hence, it is independent of the couplings between the electrons. The results show that the SE efficiency is dependent on the electron–nuclear dipolar coupling. In a simple electron–nuclear spin system, the mixing of the energy levels results in effective irradiation of the ZQ and DQ transitions that is linearly proportional to ω_*D*_^*en*^.^34^ We have previously shown that the distance dependence in SE is removed in case the electron spin is the main source of nuclear relaxation.^35^ In these simulations, for the sake of simplicity, we fixed the electron and nuclear relaxation parameters for all configurations. This, along with the absence of spin diffusion, results in the observed *r*_*en*_ dependence.

In contrast, the efficiency of the isotropic interaction-driven CE (Figure 4b bottom) decreases rapidly with the interelectron distance. In the short *r*_*ee*_ regime (under 10 *Å*), a strong ω_*D*_^*ee*^ allows transfer of polarization to significantly distant nuclei. For weaker ω_*D*_^*ee*^, CE DNP is efficient only for strongly coupled nuclear spins. These results are consistent with the CE efficiency relying on efficient state mixing which is proportional to ω_*D*_^*ee*^ · ω_*D*_^*en*^ leading to transfer of spin populations.^34^

To correlate the simulated results and the experimental measurements, it is necessary to assess the regime of interdopant distances in our systems. Assuming a homogeneous dopant distribution, the interdopant average distance, *r̅*_*ee*_, can be estimated by Wigner–Seitz model as^36^

6 $${r®}_{ee}=2\cdot {\left(\frac{3}{4\pi {\mathrm{CN}}_{A}}\right)}^{1/3}$$

with *C* being dopant concentration and *N*_A_- Avogadro’s number. The *r̅*_*ee*_ for our 40 mM, 80 mM and 160 mM Mn­(II) doped samples is 43 Å, 34 Å and 27 Å, respectively. These *r̅*_*ee*_ parameters fall within the low ω_*D*_^*ee*^ regime, where only a fraction of the dopants in the sample contribute to the signal enhancement via the CE mechanism (see Figure 4b). These results suggest that to increase the CE efficacy, we must increase the dopant concentration further. However, this comes at a cost of increasing the fraction of undetectable nuclei (which would fall within the quenching sphere of the dopants), and with further concentration increase, a decrease in electron relaxation times is expected. The later would lower the microwave saturation efficiency.^31^ Indeed, the efficiency of the CE was increasing with dopant concentration for LTO while it remained roughly constant for NZPO.

To gain further insights into the low CE efficiency in NZPO, despite it being seemingly closer to matching the CE condition (*δ*_*NZPO*_ = 2 *MHz*, compared to LTO *δ*_*LTO*_ = 4 *MHz*), we turn to examine the coordination environment of Mn­(II). Our simulations (Figure 4b) indicate that for realistic ω_*D*_^*ee*^ only the first few coordination shells of nuclei surrounding the dopant can be polarized by the CE mechanism. To this end, we performed electron–nuclear double resonance (ENDOR) experiments on a W-band system which allows probing the interactions between Mn­(II) and the coupled, ^7^Li and ^31^P nuclear spins surrounding it.^37^ Figure 5a shows the ENDOR results for the 40 mM Mn­(II) doped LTO (top) and NZPO (bottom) samples along with fits obtained with EASYSPIN.^29^ The *e*_*Mn*_-^7^Li Mims ENDOR spectrum displays two distinct dipolar Pake patterns that can be fitted with *r*_*en*_ of 3.7 and 5.7 *Å*. The *e*_*Mn*_-^31^P Davies ENDOR, on the other hand, exhibits a doublet that can be fitted with through-bond isotropic interactions of 5 MHz (along with dipolar broadening). Hence, this is the expected frequency shift for core ^31^P nuclei in a Mn–O–P coordination. The difference in the nature of the coupling interactions originates from the higher covalency of the Mn–O–P bonds compared to Mn–O–Li bonds, which are commonly more ionic. While both through-space and through-bond interactions shift the spin energy levels, in the isotropic case, this shift is constant and independent of MAS. This leads to an actual larger mismatch of the CE condition in the NZPO system. The simulated dependence of the CE efficiency on the magnitude of the deviation from the CE matching condition (δ = Δ*ω*_*ee*_ – |*ω*_*n*_|) is plotted in Figure 5b for realistic dipolar coupling of ω_*D*_^*ee*^ = 15 *MHz* (*r*_*ee*_ = 15 *Å*) and ω_*D*_^*en*^ = 1.14 *MHz* (*r*_*en*_ = 3 *Å*). The simulations show a large drop in CE efficiency with increasing |δ|.

**Figure 5:**
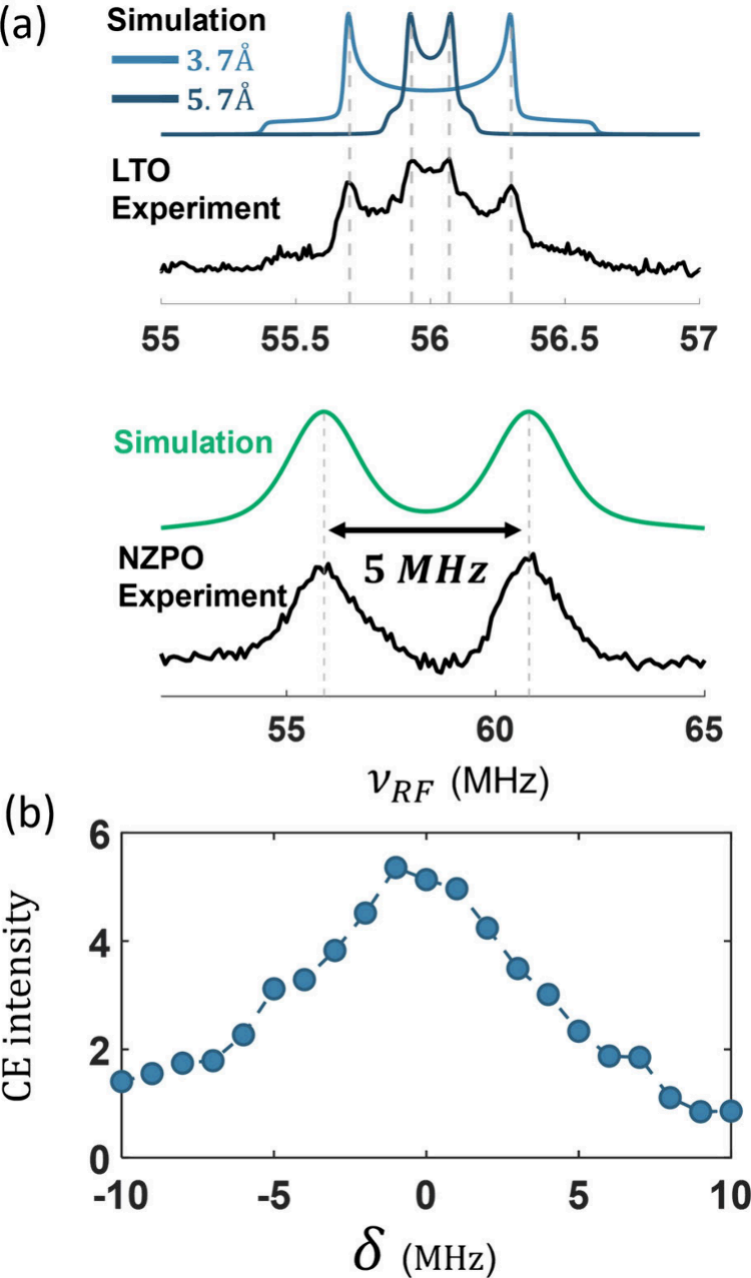
(a) ^7^Li (top) and ^31^P (bottom) ENDOR spectra (black) measured at the W-band at 8 K for the 40 mM doped LTO and NZPO samples. Simulation of the spectra for ^7^Li (two sites, blue) and ^31^P (green). (b) CE enhancement simulated for e-e-n system with different matching of the CE condition (δ = Δ*ω*_*ee*_ – *ω*_*n*_), simulated parameters: ω_*D*_^*ee*^ = 15 *MHz* (*r*_*ee*_ = 11 Å) and ω_*D*_^*en*^ = 1.14 *MHz* (*r*_*en*_ = 3 *Å*), *T*_1*e*_ = 1 *μs*, *T*_2*e*_ = 50 *ns*, *T*_1*n*_ = 100 *s*, *T*_2*n*_ = 1 *ms*.

These results could explain the relatively low CE efficiency measured in NZPO. The core ^31^P nuclei, which seem to be the most relevant for the CE at the Mn concentrations studied here, have a larger frequency offset compared to that of the bulk nuclei at 14.1 T. Consequently, the CE mechanism for these critical nuclei is ineffective. Furthermore, the large isotropic Fermi contact shift of these core nuclei would likely hinder spin diffusion between them and bulk nuclei. The reduced efficiency of spin diffusion will also limit the transfer of polarization originating from the SE mechanism. These insights explain the overall low enhancement observed in the NZPO system.

Another key factor that may influence this mechanism is the relaxation rate of the polarizing agent. Spin–lattice relaxation time, *T*_1*e*_, plays a critical role in the efficiency of the conventional anisotropic CE mechanism. As previously noted, the microsecond-scale *T*_1*e*_ commonly observed in paramagnetic metal ions is a major reason that the classic anisotropic CE is generally inefficient for such polarizing agents. Simulations of the SE and CE contribution as a function of the electron relaxation are shown in the Supporting Information (Figure S7). As expected, SE displays very strong dependence on the relaxation while CE is increasing with electron relaxation up to an enhancement factor of ∼ 100 at about 70 *μs* but remains less effective than the simulated SE. Measurements performed at low field (3.4 T) and low temperature (4 K) reveal the Mn­(II) *T*_1*e*_ is 75 and 76 μs for LTO and NZPO, respectively. This value is likely an overestimate of the relaxation at 100 K, which can also be evaluated from the PRE effect.^31,38^ Based on the ^7^Li and ^31^P nuclear relaxation times we can estimate the Mn­(II) relaxation as 1 and 0.5 μs, respectively (see Supporting Information for details). Simulations of the microwave amplitude dependence performed at short polarization time (1 ms), for which the CE is the dominant DNP mechanism, showed that the CE enhancement grows by 2 orders of magnitude in the microwave nutation frequency range of 0.1–10 MHz (Figure S9). Thus, we conclude that electron spin relaxation might also be limiting in terms of the ability to saturate the electron transition, but not more than it is for SE. This simulated result does not match the experimental observation (Figure 3b). The discrepancy may be due to sample heating which is not taken into account in simulations.

Finally, we must also consider the probability that two closely coupled electrons occupy adjacent EPR transitions, which is necessary to satisfy the CE condition. As illustrated by the field sweep profile (Figure 2a), only the outermost transitions contribute to an efficient CE enhancement with all other configurations either resulting in signal cancellation due to opposing mechanisms or failing to meet the CE matching condition. Given that the manifold of hyperfine transitions splitting the central electron spin transition in Mn­(II) are equally likely to be populated, the probability for a pair of electrons fulfilling the CE condition is only ∼ 0.6%. This low probability represents a significant intrinsic limitation on the efficiency of the isotropic CE mechanism in Mn­(II)-doped systems.

In conclusion, we demonstrated the feasibility of CE driven by isotropic interactions in MIDNP. We showed that this mechanism is independent of MAS frequency, enabling CE enhancement even with polarizing agents that exhibit short electron spin relaxation times such as paramagnetic metal ions. This approach can potentially be used for probing low-concentration chemical environments, buried interfaces, and nuclei that otherwise produce weak NMR signals while being compatible with the high resolution provided by fast MAS.

In the manganese-doped systems presented here, the main limiting factors for CE enhancement are (i) deviation from the CE conditions – which is more pronounced in covalent system prone to Fermi contact interactions, (ii) the low probability of strongly coupled electron pairs, which can be increased by higher dopant concentration, (iii) fast electron relaxation, and (iv) the low probability of populating the two outermost adjacent electron spin hyperfine levels in the central transition manifold, required to match the CE condition. We have also found that the core nuclei, directly coupled to the metal ions, are the most likely to undergo CE. Yet as these nuclei are typically undetectable, efficient distribution of CE polarization in the bulk requires significant spin diffusion. We expect this mechanism can be met more easily and diversly on DNP-NMR spectrometers that have a broader range of field sweep compared to the commercial system used here.^39^ This can enable more flexible matching of the nuclear Larmor frequency to Mn­(II) hyperfine coupling. Such flexibility may also expand the range of materials and nuclei that can be explored by achieving a CE DNP driven by isotropic interactions. With the current commercially available DNP-NMR systems, we anticipate that CE DNP based on isotropic interactions can also be realized in other lithium oxides and phosphates, using comparable manganese dopant concentrations and DNP setup, since the isotropic hyperfine parameters are largely determined by the dopant’s local chemical environment.

We predict that advancing pulse DNP techniques, which could redistribute the electron spin populations across the hyperfine manifolds (as well as satellite transitions), is an interesting path to explore for enhancing the efficiency of isotropic CE. Alternatively, rational design of polarizing agents that combine isotropic hyperfine coupling with nuclear spin lower than 5/2 and tunable electron–electron distance may provide a powerful route toward achieving robust and more efficient isotropic CE DNP enhancement.

## Supplementary Material



## Notes

Additional NMR and DNP experiments, simulations and information: Full CE DNP Hamiltonian, sample preparation, X-ray diffraction and EPR characterization, full simulation parameters, simulations of DNP buildup curves, simulations of DNP dependence on electron relaxation and microwave amplitude, DNP buildup measurements and relaxation measurements (PDF)
