# Supplementary material for: Isotropic Hyperfine Interactions Drive Cross-Effect Dynamic Nuclear Polarization
Source: J Phys Chem Lett. 2025 Nov 7;16(46):11955–63. doi: 10.1021/acs.jpclett.5c02845 (PMC12641482; doi:10.1021/acs.jpclett.5c02845)
Supplement: Supplementary file 1 [file jz5c02845_si_001.pdf]

Supporting Information

for

Isotropic Hyperfine Interactions Drive Cross-Effect Dynamic Nuclear Polarization

Nitzan Livni[(1)], Subhradip Paul[(2,3)], Ilia B. Moroz[(1)], Alexey V. Bogdanov[(4)], Daniel Jardón-Álvarez[(1)], Frederic Mentink-Vigier[(5)], Michal Leskes*[(1)]

[(1)]Molecular Chemistry and Materials Science, Weizmann institute of science, Rehovot, 761000, Israel

[(2)]Nottingham DNP MAS NMR Facility, University of Nottingham, Nottingham NG72RDEngland, UK.

[(3)]Univ. Grenoble Alpes, CEA, IRIG-MEM, 38000 Grenoble, France

[(4)]Chemical and Biological Physics, Weizmann institute of science, Rehovot, 761000, Israel

[(5)]National High Magnetic Field Laboratory, Florida State University, 1800 E Paul Dr, Tallahassee, FL, 32310, USA

*michal.leskes@wizmann.ac.il

Table of Content

1. Cross-effect dynamic nuclear polarization Hamiltonian 2

1.1. Anisotropic driven cross-effect DNP 2

1.2. Isotropic driven cross-effect DNP 3

2. Sample preparation 3

2.1. Mn(II) doped $Li_4Ti_5O_{12}$ 3

2.2. Mn(II) doped $Na_2ZnP_2O_7$ 4

3. X-ray diffraction measurements 4

4. Mn(II) electron paramagnetic resonance measurements 4

4.1. CW-EPR measurements 5

4.2. ED-EPR measurement 6

5. DNP-ssNMR measurements 6

6. DNP-MAS-NMR Simulations 6

6.1. Projection on [-1/2,1/2] Subspace 7

6.2. Polarization buildup simulations 7

7. DNP-MAS-NMR measurements 9

7.1. Polarization buildup measurements 9

8. Limiting factors of the CE mechanism 11

8.1. The effect of electron relaxation 11

8.2. Microwave amplitude dependence 12

9. Electron relaxation determined from NMR experiments 13

9.1. Nuclear spin relaxation measurements 13

9.2. T_{1e} calculation 14

10. Probability of matching the CE condition 14

References 15

1. Cross-effect dynamic nuclear polarization Hamiltonian

1.1. Anisotropic driven cross-effect DNP

The simplest model for describing the anisotropic driven cross-effect DNP mechanism requires a three-spin system: two coupled electron spins and one nuclear spin, coupled to one of the electrons. This simplified system can be described by a Hamiltonian which contains the Zeeman interactions ($\hat{H}_z^i$) of each spin, the couplings between them ($\hat{H}_D^{ij}$) and the microwave term ($\mathcal{H}_{\mu W}$). In the electron spins rotating frame, this Hamiltonian can be written as:[1]

$$\mathcal{H}_0 = \mathcal{H}_z^{e_1} + \mathcal{H}_z^{e_2} + \mathcal{H}_z^{n} + \mathcal{H}_{HF}^{en} + \mathcal{H}_D^{ee} + \mathcal{H}_{\mu W} \tag{1}$$

Where the electron spin Zeeman interactions are:

$$\mathcal{H}_z^{e_i} = (\frac{g_i \mu_B}{\hbar} B_0 - \omega_{\mu W})\hat{S}_z^i \tag{2}$$

μ_B is the electron Bohr magneton, $\hbar$ is the reduced Planck constant, B_0 is the external magnetic field, g_i is the electron g-factor, ω_{MW} is the applied microwave frequency and $\hat{S}_z^{\mathrm{i}}$ is the electron spin's angular momentum operator along the z axis. If the system contains g-anisotropy, with magic angle spinning, g_i becomes time dependent. This is commonly the main interaction that contributes to the periodic change in energy levels that facilitates the CE mechanism.

The nuclear spin Zeeman interaction:

$$\mathcal{H}_z^n = -\omega_n \hat{I}_z \tag{3}$$

With ν_n being the Larmor frequency and $\hat{I}_z^n$ is the nuclear spin's angular momentum operator along the z axis.

The pseudo secular electron-nuclear hyperfine interactions:

$$\mathcal{H}_{HF}^{en} = \sum_i \left\{ A^i \hat{S}_z^i \hat{I}_z + \frac{1}{2}\left(B_+^i \hat{S}_z^i \hat{I}_+ + B_-^i \hat{S}_z^i \hat{I}_- \right) \right\} \tag{4}$$

With A_z^i, B_+^i, B_-^i are the interaction coefficients, and $\hat{I}_+$ and $\hat{I}_-$ are the raising and lowering operators for the nuclear spin, respectively.

The secular electron-electron dipolar interactions:

$$\mathcal{H}_{D/J}^{ee} = J\hat{S}_z^1 \hat{S}_z^2 + D\left(2\hat{S}_z^1 \hat{S}_z^2 - \frac{1}{2}\left(\hat{S}_+^1 \hat{S}_-^2 + \hat{S}_-^1 \hat{S}_+^2 \right) \right) \tag{5}$$

Where J is the magnitude of the exchange interaction and D is the magnitude of the through-space dipolar couplings. $\hat{S}_+^i$ and $\hat{S}_-^i$ are the raising and lowering operators for the electron spin, respectively. These last two interactions also become time dependent upon spinning. Lastly, the microwave Hamiltonian:

$$\mathcal{H}_{\mu W} = \omega_1 \sum_i S_x^i$$

Where ω_1 is the microwave nutation frequency $\hat{S}_x^i$ is the electron spin's angular momentum operator along the x axis.

1.2. Isotropic driven cross-effect DNP

To facilitate isotropic driven cross-effect DNP we need to include the metal ions' nuclear spins with the dominated isotropic hyperfine interactions ($\mathcal{H}_{\mathrm{MIHF}}^{en}$). The anisotropic hyperfine interactions between the electron spin and nuclear spin of the metal ion are averaged out and can be ignored. We also neglect the quadrupole interaction of the detected nuclei in our system. We assume there are no other strong anisotropic interactions. For paramagnetic metal ions, those will mostly be the g-anisotropy and zero-field splitting. Therefore, the Hamiltonian of such system can be written as:

$$\mathcal{H}_0 = \mathcal{H}_z^{e_1} + \mathcal{H}_z^{e_2} + \mathcal{H}_z^{n} + \mathcal{H}_{HF}^{en} + \mathcal{H}_D^{ee} + \mathcal{H}_{\mathrm{MIHF}}^{en}, \tag{6}$$

where the electron spin Zeeman interactions are the same as above except, they have minimal or no time dependence upon spinning. The nuclear spins Zeeman interaction:

$$\mathcal{H}_z^n = -\sum \omega_n^j \hat{I}_z^j, \tag{7}$$

with the sum is over all three nuclei in the system. The electron-detected nuclear hyperfine interactions and electron-electron dipolar interactions are the same as in equations 4 and 5. The metal-ion isotropic hyperfine interactions:

$$\mathcal{H}_{MIHF}^{en} = \sum_i A_{MI}^i \hat{S}_z^i \hat{I}_z^i, \tag{8}$$

where the sum is only over the metal-ion nuclear spins in the system.

2. Sample preparation

2.1. Mn(II) doped $Li_4Ti_5O_{12}$

$Li_4Ti_5O_{12}$ (LTO) doped with Mn(II) was prepared by solid state synthesis as previously described.[2] Three samples with varying Mn(II) concentrations were prepared and studied: 40mM (0.2 w%), 80mM (0.4 w%) and 160mM (0.8 w%). Samples were characterized using powder X-ray diffraction (pXRD, Figure S1) and EPR spectroscopy (Figure S2).

2.2. Mn(II) doped $Na_2ZnP_2O_7$

$Na_2ZnP_2O_7$ (NZPO) doped with Mn(II) samples were prepared by solid state synthesis. ZnO (Alfa Aesar, 99.99% purity), Na_2CO_3 (Sigma-Aldrich, ≥99.5% purity) and $NH_4H_2PO_4$ (Arcos organics, 99.999% purity) were weighed according to mole ratio (1:1:2) and mixed with $MnCO_3$ (Alfa Aeser, 99.9% purity) using ball milling for 12 min. The powder was placed in alumina boat and calcined in a tubular oven at 300 C for 1 h, 600 C for 1 h, and 720 C for 21 h in N_2 flow (with 5 min ramp for all steps). Two samples with varying Mn(II) concentrations were prepared and studied: 40 mM (0.14 w%) and 80 mM (0.28 w%). Samples were characterized using pXRD (Figure S1) and EPR spectroscopy (Figure S2).

3. X-ray diffraction measurements

PXRD was performed using a ULTIMA-III Rigaku diffractometer operating at 40 kV and 40 mA. XRD patterns were collected between 10-120° at a scanning rate of 1 °/min (S1). The phase analysis of the NZPO samples showed the sample yield was 100% with no detectable impurities. For the LTO system, phase analysis yielded the following results: for the 40mM Mn(II), $82.1 \pm 0.3\ wt\%$ is the spinel $Li_4Ti_5O_{12}$ phase with the remaining $17.9 \pm 0.5\ wt\%$ is an impurity phase Li_2TiO_3. For both 80mM and 160mM Mn(II), $89 \pm 3\ wt\%$ is the spinel $Li_4Ti_5O_{12}$ phase with the remaining $11 \pm 1\ wt\%$ an impurity phase Li_2TiO_3.

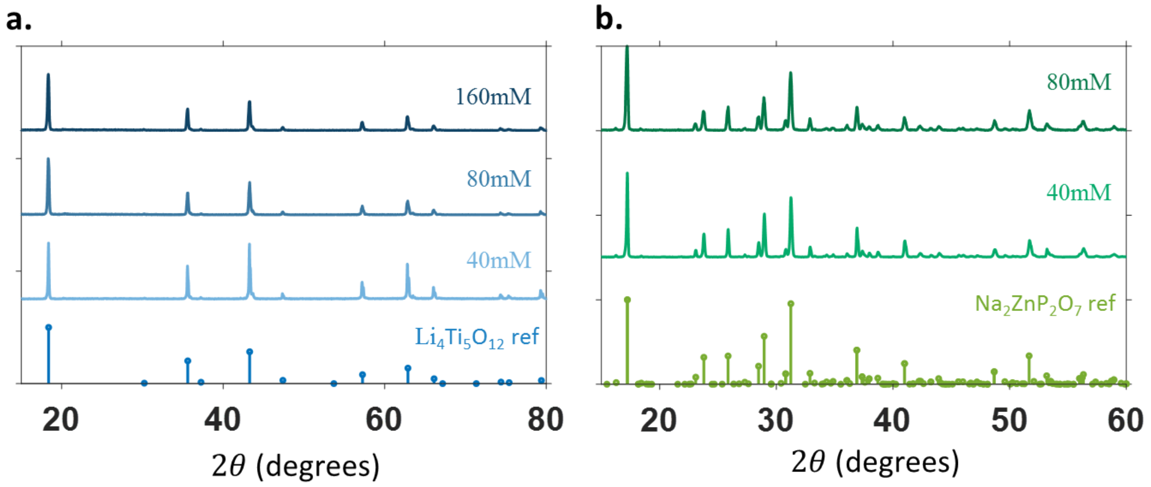

Figure S1. Powder x-ray diffraction profile of Mn(II) doped **a.** LTO and **b.** NZPO show successful synthesis of the two materials.

4. Mn(II) electron paramagnetic resonance measurements

Continuous wave (CW) EPR measurements were performed using Bruker ESR5000 spectrometer using 80 mW microwave power at RT.

Pulsed EPR and ENDOR measurements were performed using a pulsed W-band EPR spectrometer built on a basis of a 0–5 T cryogen free magnet with integrated variable temperature unit and $\mp 300 mT$ sweep coil (J3678, Cryogenic Ltd.)[3] upgraded to allow for ENDOR measurements using a cylindrical TE_{011} cavity and Helmholtz RF coil as described earlier.[4] It is equipped with 2 W pulsed microwave power amplifier (QPP95023330-ZW1, Quinstar) and a pulsed radiofrequency (RF) amplifier (1 kW, 3446 Herley-AMT).

Echo-detected electron paramagnetic resonance (ED-EPR) spectra were recorded using Hahn echo ($\pi/2 - \tau - \pi - \tau -$ echo) sequence with $\tau = 500$ ns. Phase memory times T_M were estimated from Hahn echo decay, using the same sequence with varied τ. Spin-lattice relaxation times T_{1e} were estimated using the inversion recovery sequence, $\pi - t_{wait} - \pi/2 - \tau - \pi - \tau -$ echo, with varying t_{wait}. ENDOR spectra were recorded at 8 K using Mims ($\pi/2 - \tau - \pi/2 - T(\pi_{RF}) - \pi/2 - \tau -$ echo $- [\tau_2 - \pi - \tau_2 -$ echo$]_n$) and Davies ($\pi - T(\pi_{RF}) - \pi/2 - \tau - \pi - \tau -$ echo $- [\tau_2 - \pi - \tau_2 -$ echo$]_n$) ENDOR sequences, using four-step phase cycles and a Carr-Purcell Meiboom-Gill (CPMG) detection train at the end for enhancing signal-to-noise ratio.[5] Random sampling of RF was employed,[6] with 5 shots acquired per point and a repetition rate of 100 shots per second.

Microwave power was adjusted to give a π pulse of 96 ns for Davies ENDOR and 28 ns for the rest of the experiments, using Rabi nutation sequence, $t_{nut} - t_{wait} - \pi/2 - \tau - \pi - \tau -$ echo (t_{nut} was varied; t_{wait} was chosen such as to let for the decay of the transverse magnetization). RF power was adjusted to yield a desired π_{RF} pulse length, using a Rabi nutation sequence $\pi/2 - \tau - \pi/2 - T(t_{RF}) - \pi/2 - \tau$ – echo, with a constant mixing time T and varying RF pulse length, t_{RF}.

4.1. <u>CW-EPR measurements</u>

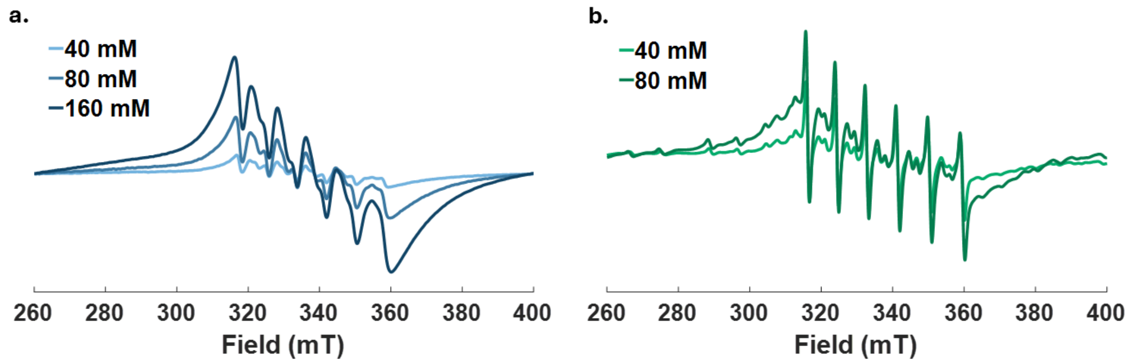

Figure S2. X-band CW EPR spectrum of Mn(II) doped **a.** LTO and **b.** NZPO showing the characteristics EPR profile of Mn(II), indicating a successful doping. Signal intensity increases with dopant concentration.

4.2. ED-EPR measurement

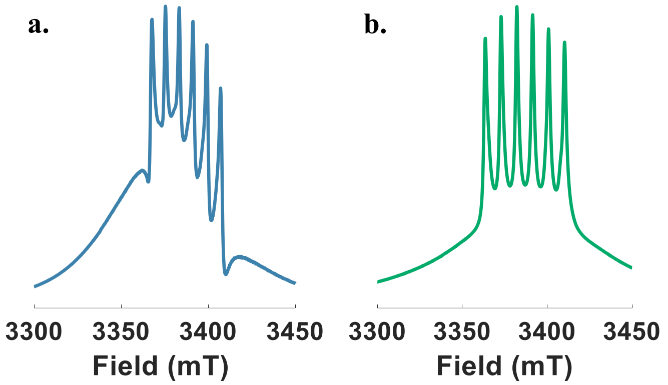

Figure S3. W-band echo detected field-sweep EPR spectrum of 40mM Mn(II) doped **a.** LTO and **b.** NZPO showing the characteristics splitting of the central EPR transition due to hyperfine interaction with the manganese 5/2 nuclear spin. Broad components are due to higher electron spin transitions. Hyperfine parameters, calculated from the distance between the peaks of the central transition, were found to be 229 MHz and 245 MHz for Mn(II) doped in LTO and NZPO, respectively.

5. DNP-ssNMR measurements

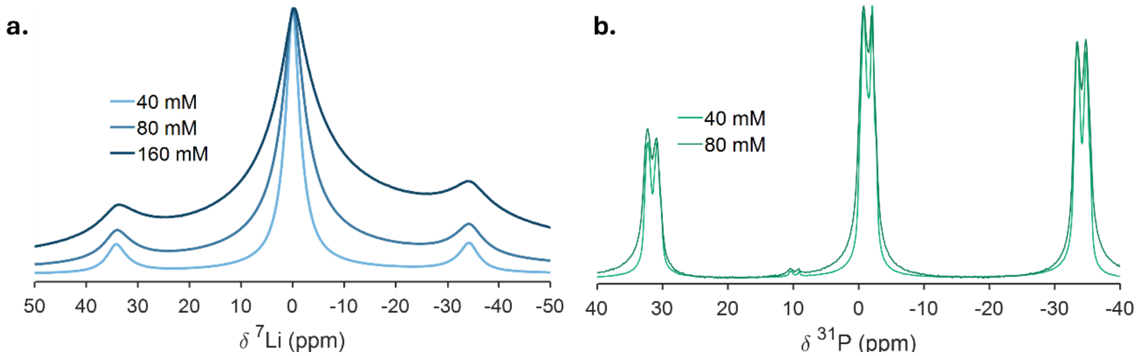

Figure S4. DNP-NMR measurements of (a) ^{7}Li in LTO and (b) ^{31}P in NZPO with different dopant concentrations. Due to the PRE effect, higher dopant concentration leads to broadening of NMR spectra, resulting in some loss of spectral resolution.

6. DNP-MAS-NMR Simulations

MAS-DNP simulations were carried out with a previously described numerical model.[1,7,8] All plots were simulated using microwave frequency of 395 GHz, microwave nutation frequency of 0.35 MHz and MAS frequency of 10 kHz at magnetic field of 14.1 T and temperature of 100 K. The nuclear Larmor frequency was set to $\omega_n = 233$ MHz, corresponding to the ^{7}Li Larmor frequency at 14.1 T. The isotropic hyperfine coupling was set to the experimentally measured value of $A^{iso} = 229$ MHz for Mn(II) in LTO. For the simulation of the full DNP sweep profile with two electrons (Fig. 2a, main text), an electron–electron dipolar coupling of 15.4 MHz was used, along with a dipolar coupling of 1.14 MHz between the nuclear spin and one of the electrons, with no coupling

to the second electron. Polarization time was 100 ms. The field sweep ranged from 14.08 T to 14.15 T with 300 linearly spaced points. The following relaxation parameters were applied in all simulations unless stated otherwise: T_{1e}= 1 μs, T_{2e}= 50 ns, T_{1n} = 100 s, and T_{2n} = 1 ms.

6.1. Projection on [-1/2,+1/2] Subspace

Simulations with spins S > ½ tend to be long as the size of the problem increases. Yet most of the DNP physical phenomena occur within [-1/2,+1/2] manifold. To account for the Zero-Field Splitting and speed up the simulations, the solution chosen here consists of projecting the Hamiltonian on the [-1/2,+1/2] subspace of each spin:

$$\hat{H}_0^p = \hat{P}\hat{H}_0\hat{P}',$$

where $\hat{P}$ is a projection matrix that only selects the energy levels $|m_{s,1}, m_{s,2}, \phi\rangle$, where $m_{s,i} \in [-1/2, +1/2]$ and ϕ is the nuclear state. This projected Hamiltonian is then used to build the Liouvillian and the super-operator propagator which have significantly reduced size. The underlying assumptions are (1) the other levels do not contribute to the DNP process, (2) the nuclear relaxation of the Mn nuclei is fast enough and has low polarization to enable decoupling the subspaces. The enhancement obtained needs to be corrected for the size reduction. For the purpose of this work, this has been implemented in the code previously described.[8]

The enhancement is then defined as

$$\epsilon^P(t) = \frac{trace\left(\hat{\rho}(t)\left(\hat{P}\hat{I}_z\hat{P}'\right)\right)}{trace\left(\hat{\rho}(0)\left(\hat{P}\hat{I}_z\hat{P}'\right)\right)},$$

Where $\hat{\rho}^p(t)$ is the density matrix at time t based on the propagation of $\hat{H}_0^p$ and $\hat{I}_z$ the Pauli operator for the nucleus.

6.2. Polarization buildup simulations

The enhancement buildup as a function of the polarization time was simulated at a field range of 14.13-14.15 T, for two scenarios: (1) Matching the CE condition ($A^{iso} = \omega_n$) exactly and (2) off CE condition with hyperfine $A^{iso} = 229$ MHz. For both, the electron-electron dipolar couplings were set to 39 MHz ($r_{ee} = 11$ Å). All other parameters were described above. The ε_{SE} and ε_{CE} were calculated according to the equations given in the main text. The buildup curves were fitted with mono or biexponential functions:

$$f(t) = A\left(1 - e^{-\frac{t}{\tau}}\right) \tag{9}$$

$$f(t) = A\left(1 - e^{-\frac{t}{\tau_1}}\right) + (1 - A)\left(1 - e^{-\frac{t}{\tau_2}}\right) \tag{10}$$

Results are presented in Figure S5. Our simulation does not include spin diffusion; hence, the buildup time corresponds directly to the underlying DNP mechanism. At the outermost DNP sweep lobe (14.1435 T), located at the DQ transition which corresponds to pure SE mechanism, the polarization buildup displays mono-exponential growth, with characteristics buildup time of ~100 ms. In contrast, at the inner sweep lobe (14.135 T), the polarization build up was fitted with biexponential function, with one characteristic buildup time in the ms and a second in the ~100 ms. These results support the conclusion that the CE DNP mechanism contributes to signal enhancement in this system. In the scenario of perfect matching of the CE condition, for the chosen simulation parameters, the time scales of the two DNP mechanisms are nicely separated i.e., the CE mechanism reaches a plateau before the SE mechanism starts to contribute to the enhancement. In the scenario of the experimental hyperfine, the CE mechanism buildup is slower, and the time scale of both mechanisms overlaps. We note that for both cases, at long polarization times, there is a slight decrease in ε_{CE}. This is an indication that the two DNP mechanisms are competing to a certain extent.

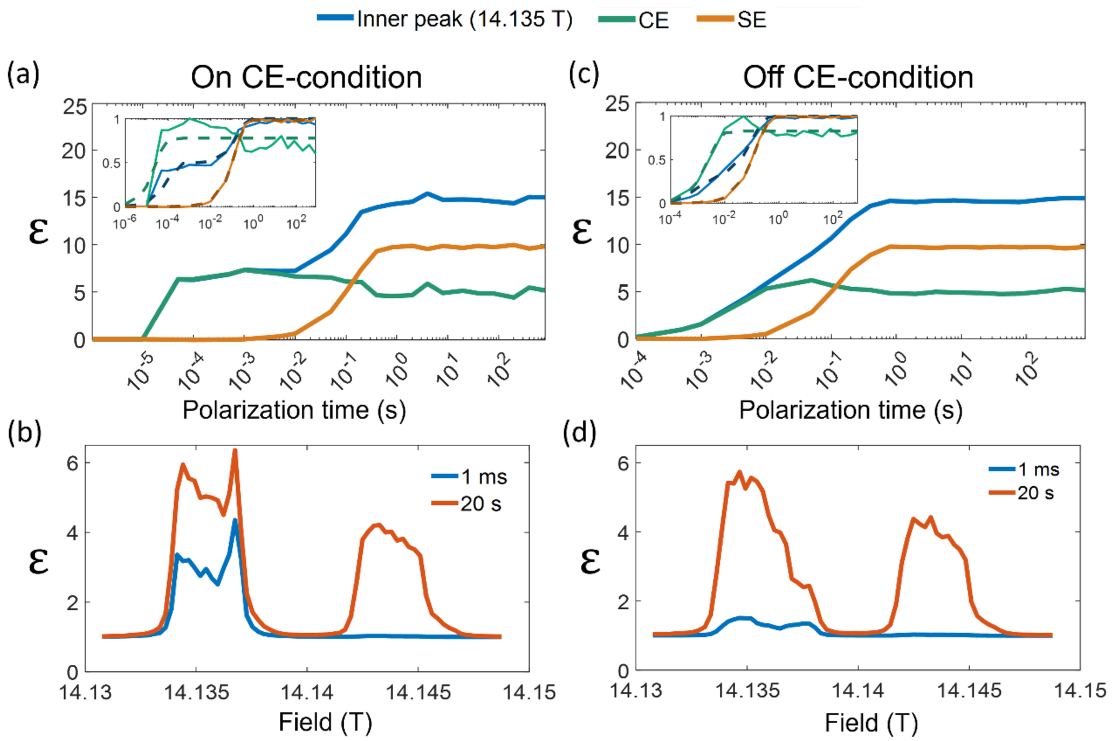

Figure S5. Simulated DNP enhancement buildup curves for the case of (a) perfect matching the CE condition ($A^{iso} = \omega_n$) and (c) realistic hyperfine ($A^{iso} = 229\ MHz$). Inserts show normalized buildup curves with fits according to equations (9) and (10). (b) and (d) are the corresponding simulated partial DNP sweep profiles at polarization time of 1 millisecond (blue) and 20 seconds (orange). At short polarization times, only the low field peak is observed, and the fast buildup of signal can be attributed to the CE mechanism.

7. DNP-MAS-NMR measurements

DNP enhanced MAS-NMR measurements were carried out at Nottingham DNP MAS NMR facility (Nottingham, UK) and at the National High Magnetic Field Laboratory (Tallahassee, USA). In both places a 14.1 T magnet and 395 GHz gyrotron equipped with a Bruker Avance III console. In Nottingham DNP MAS NMR Facility a 3.2 mm MAS-DNP probe was used. In the National High Magnetic Field Laboratory, measurements were carried out with the probe based on Dubroca T. et al. work.[9] All experiments were carried out at about 100 K. Unless stated otherwise, experiments were performed with spinning frequency of 8 kHz.

7.1. Polarization buildup measurements

^{7}Li and ^{31}P magnetization buildup curves were measured using a saturation recovery experiment with microwave irradiation. The curves were fitted with a mono exponential recovery according to equation 9. All fit parameters are given in Table S1. The measurements were performed at two field positions: at the pure SE peak of the DNP field sweep profile and at the mixed DNP mechanisms peak.

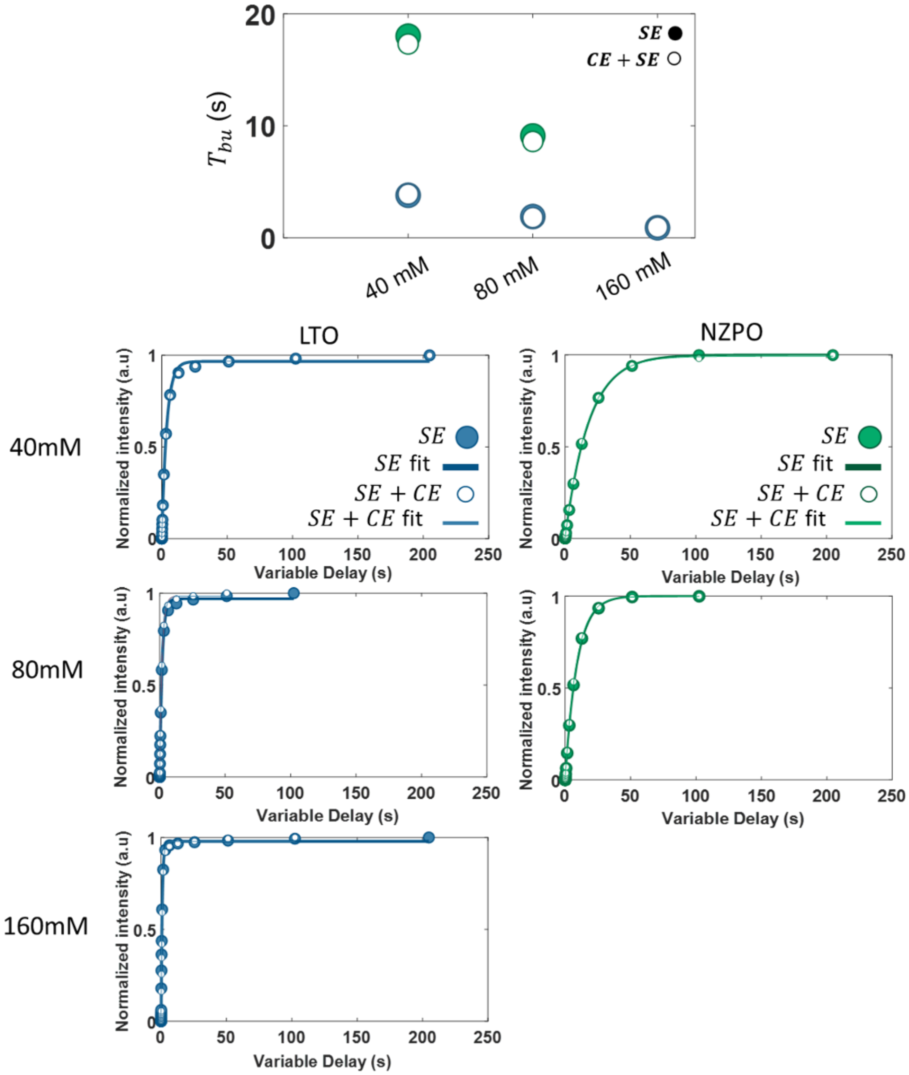

Figure S6. ^{7}Li (blue) and ^{31}P (green) magnetization buildup curves measured at two field positions in the DNP profile: pure SE peak (full dots) and at the mixed DNP mechanisms peak (empty dots). Fits (solid lines) obtained with a mono-exponential recovery according to equation (9).

Table S1. Fit parameters for ^{7}Li and ^{31}P magnetization buildup measurements

System	Dopant concentration	DNP peak	T_{bu} (s)
LTO	40mM	SE	3.8 ± 0.1
		SE+CE	3.9 ± 0.1
	80 mM	SE	1.90 ± 0.06
		SE+CE	1.80 ± 0.06
	160mM	SE	0.90 ± 0.03
		SE+CE	0.95 ± 0.04
NZPO	40mM	SE	18.1 ± 0.3

		SE+CE	17.3 ± 0.3
	80mM	SE	9.1 ± 0.2
		SE+CE	8.6 ± 0.2

8. Limiting factors of the CE mechanism

8.1. The effect of electron relaxation

The effect of longitudinal electron relaxation (T_{1e}) on the enhancement obtained from the two mechanisms is shown in Figure S7. The calculation was performed with exact matching of the CE condition ($\delta = 0$), microwave amplitude of 0.35 MHz and polarization time of 100s. The dipolar couplings used in this simulations where $\omega_D^{ee} = 39$ MHz ($r_{ee} = 11$ Å) and $\omega_D^{en} = 1.14$ MHz ($r_{en} = 3$ Å), with T_{2e} set to be $\frac{T_{1e}}{10}$. The SE mechanism shows substantial increase with longer electron spin relaxation, reflecting the higher efficiency of saturating the forbidden electron spin transitions with a slower relaxation process. The CE efficiency remains limited to single-digit enhancement when the electron spin-lattice relaxation time is shorter than 10 µs, which is typical for paramagnetic metal ions. When T_{1e} exceeds this range, the CE efficiency reaches a maximal enhancement of approximately $\varepsilon_{CE} \approx 100$ for $T_{1e} = 70\ \mu$s. Overall, the observed enhancement remains significantly lower than that typically achieved using organic radicals with comparable dipolar couplings and relaxation times.

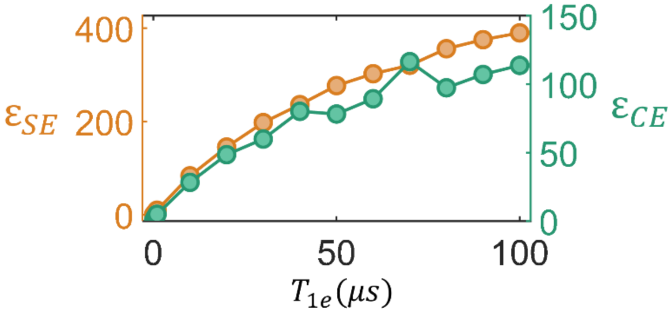

Figure S7. T_{1e} effect on DNP efficiency. Simulated SE (orange) and CE (green) enhancement with T_{1e} changing from 0 to 100 μs and $T_{2e} = \frac{T_{1e}}{10}$. Simulations performed with exact matching of the CE condition ($\delta = 0$). The dipolar couplings used in these simulations were $\omega_D^{ee} = 39$ MHz ($r_{ee} = 11$ Å) and $\omega_D^{en} = 1.14$ MHz ($r_{en} = 3$ Å).

Next, we turn to examine the effect of transverse electron relaxation (T_{2e}) on the DNP mechanisms in the system. Figure S7 shows ε_{SE} and ε_{CE} that were obtained from simulation of the DNP sweep

with varying T_{2e} parameters and fixed $T_{1e} = 1\ \mu s$.The rest of the simulation parameters are identical to those mentioned for the above simulations.

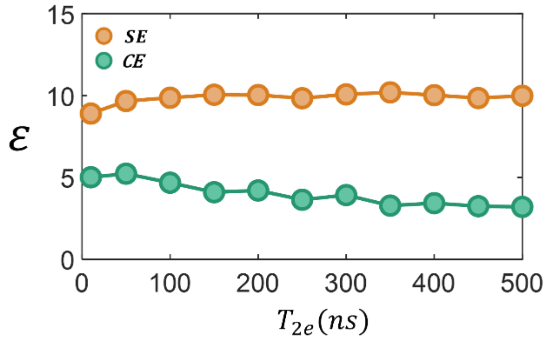

Figure S8. T_{2e} effect on DNP efficiency. Simulated SE (orange) and CE (green) enhancements with T_{2e} in the range of 10 to 500 ns and $T_{1e} = 1\ \mu s$. Simulations performed with exact matching of the CE condition ($\delta = 0$). The dipolar couplings used in these simulations were $\omega_D^{ee} = 39$ MHz ($r_{ee} = 11$ Å) and $\omega_D^{en} = 1.14$ MHz ($r_{en} = 3$ Å).

8.2. Microwave amplitude dependence

The microwave amplitude dependence of the CE and SE mechanisms in a case of perfect matching of the CE condition ($A^{iso} = \omega_n$) was simulated in a range of 0.1 to 10 MHz at two polarization time: 100 s and 1 ms. The dipolar coupling and relaxation parameters used for this calculation were: $\omega_D^{ee} = 39$ MHz ($r_{ee} = 11$ Å) and $\omega_D^{en} = 1.14$ MHz ($r_{en} = 3$ Å), $T_{1e} = 1\ \mu s, T_{2e} = 100\ ns, T_{1n} = 100\ s, T_{2e} = 1\ ms$. The results are presented in Figure S9.

At long polarization time (full symbol), when the enhancement buildup of both mechanisms reached a plateau (see Figure S5), the SE shows strong microwave amplitude dependence, with ε_{SE} being two times higher than ε_{CE} at realistic microwave amplitude (up to 1 MHz) and a few orders of magnitude larger than ε_{CE} at higher powers. In contrast, at long polarization time, the microwave amplitude has low impact on the CE enhancement. For short polarization time (empty marks), the situation is different. SE enhancement is negligible, even at high microwave amplitude. The CE mechanism however displays steady power dependence. Interestingly, ε_{CE} at short polarization time is shown to be higher than ε_{CE} at long polarization time. This is yet another indication that the two DNP mechanisms are not independent, and that the overall enhancement at a field position where both can occur is not a pure sum of the maximum enhancement of each mechanism.

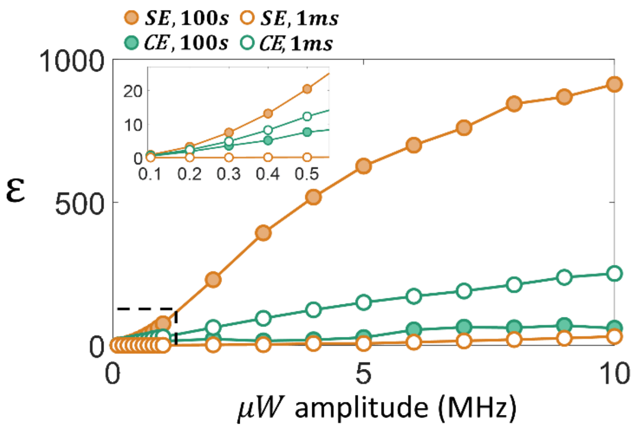

Figure S9. The effect of microwave amplitude on DNP efficiency. Simulated SE (orange) and CE (green) enhancement with microwave increasing from 0.1 to 10 MHz. The power dependence was simulated for polarization time of 100s (full dots) and 1ms (empty dots). Simulations performed with exact matching of the CE condition ($A^{iso} = \omega_n$) ,dipolar couplings $\omega_D^{ee} = 39$ MHz ($r_{ee} = 11$ Å) and $\omega_D^{en} = 1.14$ MHz ($r_{en} = 3$ Å), and relaxation parameters of $T_{1e} = 1\ \mu s, T_{2e} = 100\ ns, T_{1n} = 100\ s, T_{2e} = 1\ ms$. Inset show the region of practical microwave amplitude of 0.1-0.5 MHz.

9. Electron relaxation determined from NMR experiments

As first described by Jaroniec et al. and later shown by us for inorganic solids, PRE theory can be used to estimate the electron longitudinal relaxation of the paramagnetic metal ion dopant from the nuclear longitudinal and transverse relaxation, according to the following equation:[10,11]

$$T_{1e} = \sqrt{\left(\frac{T_{1n}}{T_{2n}} - \frac{7}{6}\right) \cdot \frac{6}{4\nu_n^2}} \tag{11}$$

9.1. Nuclear spin relaxation measurements

^{7}Li and ^{31}P longitudinal relaxation curves were measured for 80 mM Mn(II) doped samples using a saturation recovery experiment without microwave irradiation. NZPO curve was fitted using a mono exponential recovery (equation 9). LTO curve was fitted using biexponential (equation 10). Transverse polarization decay times were measured with the Hahn echo sequence with varying echo delays and fitted by stretched exponential decay:

$$f(t) = e^{-\left(\frac{t}{\tau}\right)^{\beta}} \tag{12}$$

All curves are shown in figure S6. Fit parameters are given in Table S2.

Table S2. Fit parameters for [7]Li and ^{31}P relaxation for 80mM Mn(II) doped LTO and NZPO, respectively.

	LTO	NZPO
T_{1n}	$1.56 \pm 0.03\ s$ (A= 0.77)<br>$80 \pm 0.6\ s$	$11.8 \pm 0.3\ s$
T_{2n}	$0.218 \pm 0.005\ ms$<br>$(\beta =\ 0.84 \pm 0.03)$	$0.94 \pm 0.01\ ms$<br>$(\beta = 0.72 \pm 0.01\ )$

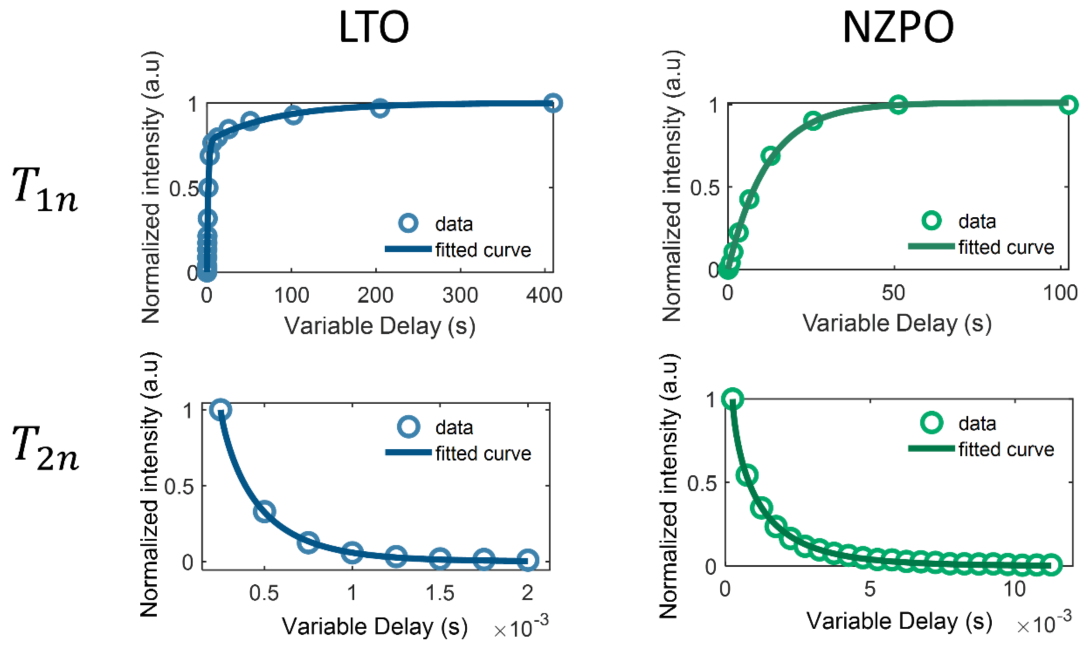

Figure S10. [7]Li (blue) and [31]P (green) relaxation curves. LTO and NZPO saturation recovery data were fitted with bi and mono exponential recovery, respectively. Transverse polarization decay curves were fitted with stretched exponential decay.

9.2. T_{1e} calculation

T_{1e} in 80 mM Mn(II) doped LTO and NZPO was calculated using equation (11). For LTO, the weighted average of the two characteristics T_{1n} was used in the T_{1e} calculation.

10. Probability of matching the CE condition

The probability of the Mn(II) electron spins to be at the central transition (CT) which is assumed to have the highest contribution to the DNP processes is 1/3. Moreover, only the spin occupying the outermost transitions out of the six SQ central transitions contribute to an efficient CE enhancement when it is coupled to an electron spin in the adjacent SQ transition. All other configurations either result in signal cancellation due to opposing mechanisms or fail to meet the

CE matching condition in the two studied systems. Given that the manifold of hyperfine transitions splitting the central electron spin transition in Mn(II) are equally likely to be populated, the probability of having the two coupled electrons in those neighboring transitions is given by:

$$P_{CE} = \left(P_{CT} \cdot \left(P_{outermost\,SQ} + P_{\text{adjacent}\,SQ}\right)\right)_{first\ electron} \cdot \left(P_{CT} \cdot P_{\text{adjacent}\,\backslash outermost\,SQ}\right)_{second\ electron}$$

When P_x is the probability for the electron to occupy each state.

Taking into account the probabilities we get that:

$$P_{CE} = \left(\frac{1}{3} \cdot \left(\frac{1}{6} + \frac{1}{6}\right)\right)_{first\ electron} \cdot \left(\frac{1}{3} \cdot \frac{1}{6}\right)_{second\ electron} = \frac{1}{164} \approx 0.6\%$$

References

1. Mentink-Vigier, F., Akbey, Ü., Oschkinat, H., Vega, S. & Feintuch, A. Theoretical aspects of Magic Angle Spinning - Dynamic Nuclear Polarization. *Journal of Magnetic Resonance* **258**, 102–120 (2015).
2. Wolf T., Kumar S., Singh H., Chakrabarty T., Aussenac F., Frenkel A. I., Major D. T. & Leskes M. Endogenous Dynamic Nuclear Polarization for Natural Abundance ^{17}O and Lithium NMR in the Bulk of Inorganic Solids. *J Am Chem Soc* **141**, 451–462 (2019).3. A. Feintuch , D. Shimon , Y. Hovav , D. Banerjee , I. Kaminker , Y. Lipkin , K. Zibzener , B. Epel , S. Vega and D. Goldfarb. A Dynamic Nuclear Polarization spectrometer at 95 GHz/144 MHz with EPR and NMR excitation and detection capabilities. *Journal of Magnetic Resonance* **209**, 136–141 (2011).
4. Gromov, I., Krymov, V., Manikandan, P., Arieli, D. & Goldfarb, D. A W-Band Pulsed ENDOR Spectrometer: Setup and Application to Transition Metal Centers. *Journal of Magnetic Resonance* **139**, 8–17 (1999).
5. Mentink-Vigier, F., Collauto, A., Feintuch, A., Kaminker, I., Tarle, V., Goldfarb D.. Increasing sensitivity of pulse EPR experiments using echo train detection schemes. *Journal of Magnetic Resonance* **236**, 117–125 (2013).
6. Epel, B., Arieli, D., Baute, D. & Goldfarb, D. Improving W-band pulsed ENDOR sensitivity - Random acquisition and pulsed special TRIPLE. *Journal of Magnetic Resonance* **164**, 78–83 (2003).
7. Can, T. V., Caporini, M. A., Mentink-Vigier, F., Corzilius, B., Walish J. J., Rosay M., Maas W. E., Baldus M., Vega S., Swager T. M., Griffin R. G.. Overhauser effects in insulating solids. *Journal of Chemical Physics* **141**, 064202 (2014).

8. Mentink-Vigier, F. Numerical recipes for faster MAS-DNP simulations. *Journal of Magnetic Resonance* **333**, 107106 (2021).

9. Dubroca, T.; Smith, A. N.; Pike, K. J.; Froud, S.; Wylde, R.; Trociewitz, B.; McKay, J.; Mentink-Vigier, F.; van Tol, J.; Wi, S *et al.* A quasi-optical and corrugated waveguide microwave transmission system for simultaneous dynamic nuclear polarization NMR on two separate 14.1 T spectrometers. *Journal of Magnetic Resonance* **289**, 35–44 (2018).

10. Mukhopadhyay, D., Nadaud, P. S., Shannon, M. D. & Jaroniec, C. P. Rapid Quantitative Measurements of Paramagnetic Relaxation Enhancements in Cu(II)-Tagged Proteins by Proton-Detected Solid-State NMR Spectroscopy. *Journal of Physical Chemistry Letters* **8**, 5871–5877 (2017).

11. D. Jardón-Álvarez, T. Malka, J. van Tol, Y. Feldman, R. Carmieli, M. Leskes. Monitoring electron spin fluctuations with paramagnetic relaxation enhancement. *Journal of Magnetic Resonance* **336**, 107143 (2022).
